# Exploring experiences of talk therapies among gay and bisexual men seeking to reduce or abstain from using crystal methamphetamine in the context of chemsex

**DOI:** 10.1111/dar.13999

**Published:** 2025-01-29

**Authors:** Jack Freestone, Bo Justin Xiao, Krista J. Siefried, Adam Bourne, Nadine Ezard, Lisa Maher, Robert Stirling, Louisa Degenhardt, Rick Varma, Garrett Prestage, Mohamed Hammoud

**Affiliations:** ^1^ The Kirby Institute UNSW Sydney Sydney Australia; ^2^ ACON Sydney Australia; ^3^ National Centre for Clinical Research on Emerging Drugs Sydney Australia; ^4^ Alcohol and Drug Service, St Vincent's Hospital Sydney Sydney Australia; ^5^ National Drug and Alcohol Research Centre, UNSW Sydney Sydney Australia; ^6^ Australian Research Centre for Sex Health and Society, La Trobe Melbourne Australia; ^7^ Burnett Institute Melbourne Australia; ^8^ Network of Alcohol and Other Drug Agencies Sydney Australia; ^9^ Drug Policy Modelling Program UNSW Sydney Sydney Australia; ^10^ Sydney Sexual Health Centre Sydney Australia

**Keywords:** health services, methamphetamine, qualitative research, sexual and gender minorities, sexuality

## Abstract

**Introduction:**

Some gay and bisexual men who have sex with men (GBMSM) who use drugs to enhance sex (chemsex/party and play) may experience harms and seek talk therapies. GBMSM who practice chemsex may not access drug services because of anticipated stigma and the perception that these services lack chemsex expertise. Barriers to services are documented, however, little is known about the service experiences of chemsex engaged GBMSM.

**Methods:**

Semi‐structured interviews were conducted with 24 participants reporting current practice of sexualised use of methamphetamine and/or gamma hydroxybutyrate. Interviews explored experiences of counselling and psychology services, participant's treatment goals and challenges. Data were transcribed verbatim and analysed in NVIVO14 with a qualitative description methodology.

**Results:**

Most in our study sought to reduce the frequency of methamphetamine use and used methamphetamine only in sexual contexts. When engaging with counsellors and psychologists in alcohol and other drug or mental health services for the general adult population, most censored the sexual drivers and types of sexual behaviours incumbent in their methamphetamine use. Participants' reliance on drugs for sex was spoken about as a major barrier to reducing methamphetamine. Sexual self‐censorship within services inhibited participants' abilities to access meaningful support and achieve treatment goals.

**Discussion and Conclusions:**

Counsellor and psychologists working with GBMSM around drug use, must ask about context of drug use and sex. Training and supervision around sexual therapies for those working alongside GBMSM who practice chemsex may be beneficial. Research on treatment approaches to support the sexual wellbeing of people who practice chemsex is required.

**Key Points**

- Gay and bisexual men who have sex with men (GBMSM) reported a treatment goal of reducing or abstaining from crystal methamphetamine use in the context of chemsex and sought service from providers of talk therapies within general adult population mental health, alcohol and other drug and sexual health services or within sexual health, alcohol and other drug and community services with chemsex expertise.
- Most GBMSM reported that they used drugs in sexual contexts and experienced a bi‐directional relationship between their drug use, sexuality and sexual behaviours.
- Many participants felt that learning to have sex without drugs would help them to reduce or abstain from chemsex.
- Most participants were not asked about sex when seeking services from counsellors or psychologists within services for the general adult population and reported barriers to disclosing sexual contexts and drivers of their drug use that were associated with anticipated stigma.
- Providers of talk therapies need to be supported to proactively ask about and address sexual wellbeing when providing services for GBMSM practicing chemsex.

## 1 INTRODUCTION

Chemsex, sometimes referred to as ‘party and play’, ‘PnP’ or ‘sexualised drug use’ has been described as the use of substances to facilitate sex that is characterised as expansive in terms of duration, partners and activities [1, 2]. Surveys conducted among a convenience sample of gay and bisexual men who have sex with men (GBMSM) in the Australian state of New South Wales indicate that 8% report use of crystal methamphetamine and 11% report use of gamma hydroxybutyrate (GHB) within the past 6 months [3]. It is estimated that over 80% of this use is for the purpose of enhancing sex [4, 5].

Although not all who practice chemsex experience harms [6, 7], a range of acute and chronic harms to both physical and mental health are apparent [1, 2]. Behavioural interventions are the first line treatment for chemsex [8] and some GBMSM who practice chemsex may require interdisciplinary talk‐therapies that address drug use, mental and sexual health [9, 10]. However, prior research conducted among non‐treatment seeking samples have indicated that GBMSM who practice chemsex experience barriers to accessing drug services and these barriers include their anticipation of stigma [11, 12, 13]. The evidence on stigma in relation to health services outlines an array of nuanced and distinct experiences that may adversely affect outcomes relating to service access, treatment retention and treatment success [14, 15]. The literature regarding chemsex among GBMSM and services has underscored experiences of anticipated stigma, a phenomenon defined as the expectation of bias being perpetrated and perceived stigma, the belief that one is, or will be, treated poorly within a given setting [16]. Prior studies indicate that GBMSM specifically anticipate and perceive service level stigmas around their use of drugs, their use of drugs for sex and the nature of sex had while using drugs [11, 12, 17, 18, 19].

In addition to concerns regarding stigma, GBMSM who practice chemsex may perceive that drug treatment services lack expertise to meet their needs, which span drug use, sexual health and sexual wellbeing [11, 20]. Several recent Australian studies among people engaged in chemsex have underscored the ways in which drugs may facilitate exploratory, gratifying and affirming sexual possibilities, sexual identities and sexual networks [6, 21, 22, 23]. Chemsex is distinct from other forms of drug use because it encompasses a bi‐directional relationship between drug use and sex [2, 21, 24, 25]. Empirical research on chemsex has highlighted risks around blood borne virus transmission [2, 26] and chemsex interventions often dually address drug use and the prevention of sexually transmitted infections [8]. The prevention of sexually transmitted infections is important, however, scholarship on sexual health has highlighted the imperative for interventions to address an array of experiences relating to sexual health and sexual wellbeing, including sexual identity, experiences of pleasure, sexual violence and sexual function [27]. Notably, several studies have reported that some GBMSM who practice chemsex are not able to have sex without drugs [28, 29, 30]. Studies among chemsex‐practicing GBMSM in the Netherlands and Spain found that 13% and 10% of their respective samples reported no recent experience of sober sex [28, 29] defined in the Dutch study as sex without the use of crystal meth, cocaine, designer drugs, GHB, ketamine, 3,4‐Methylenedioxymethamphetamine, mephedrone, speed and ecstasy [28]. GBMSM reporting no recent sober sex also report a higher desire to receive professional counselling services [28] and upon accessing services these GBMSM often aim to reengage with sober sex [31, 32].

There has been little research conducted to explore the service experiences of GBMSM engaged in chemsex and there are few theory‐based and data‐driven interventions to guide approaches to support chemsex practicing GBMSM to reengage with sex without drugs [31]. It is unknown whether services offer appropriate support to GBMSM around the bi‐directional relationship between drug use and sex. Drawing on recommendations that chemsex support services provide interdisciplinary care [20, 33, 34] our study aimed to explore how those who practice chemsex, experience services provided by counsellors and psychologists across domains of drug use and sex. We outline how participants described their service experiences in relation to their goal of reducing methamphetamine use.

## 2 METHODS

All participant names are reported as pseudonyms, this study received ethical approval from UNSW Human Research Ethics Committee, approval number HC210304.

### 2.1 Participant recruitment

Eligible participants were those who identified as GBMSM (cis and trans), transgender women and non‐binary people, living in New South Wales and reporting sexualised use of crystal methamphetamine and/or GHB within the previous 3 months. Participants were recruited via targeted advertising on ACON's social media channels and internet hookup apps and websites for GBMSM (Grindr, Recon). ACON is a community health organisation in New South Wales, Australia that specialises in health promotion, service delivery, advocacy and research for sexuality and gender diverse populations. In addition to online recruitment, participants were also referred to the study website by GBMSM chemsex peer‐workers employed by ACON or by counsellors working at the St Vincent's Hospital Stimulant treatment program. Most participants in this study were referred via ACON's chemsex peer workers.

### 2.2 Data collection

Post consent, community participants completed a survey intended to characterise the sample. This survey collected participant's demographic and behavioural characteristics, their chemsex practices and a history of their service use, service engagement was then discussed in semi‐structured interviews. As outlined in Table 1, most participants (*n* = 16) reported prior engagement with services.

**TABLE 1:** Participant characteristics (*n* = 24).

Gender	Cisgender male 22	Trans male 1	Non‐binary 1		
Sexuality	Gay 21	Bisexual 3	Queer 1		
Age, years	18–29 2	30–39 4	40–49 8	50–59 4	60+ 6
Ethnicity	Caucasian 21	Asian 3	Latinx 1		
HIV status	Negative 18	Positive 6			
Employment	Employed 20	Unemployed 4			
Use of crystal methamphetamine in last 3 months	Yes 24				
Severity of Dependence Scale for crystal methamphetamine use ≥ 4	Yes 18	No 6			
Injected crystal methamphetamine in last 3 months	Yes 19	No 5			
Frequency of crystal methamphetamine use in last 3 months	Most days 5	At least weekly 5	At least monthly 9	Less than monthly 5	
Used GHB in the last 3 months	Yes 15	No 9			
Frequency of GHB use among those who used in last 3 months	At least weekly 4	At least monthly 6	Less than monthly 3	Once 2	
Ever use of counselling or psychologist services for drug support				*n* = 16^a^	
Specialist services				*n* = 14^b^	
ACON Substance Support Counselling Service				*n* = 5	
St Vincent's Stimulant Treatment Program (specialist drug and alcohol service)				*n* = 6	
Counselling via a publicly funded sexual health service				*n* = 7	
Other LGBTQ+ drug counselling service				*n* = 1	
Mainstream services				*n* = 12	
Outpatient counselling or psychologist service for the general adult population					
No use of counselling or psychologist service for drug support				*n* = 8	

Abbreviation: GHB, gamma hydroxybutyrate; LGBTQ, lesbian, gay, bisexual, transgender, queer.

^a^ Ten participants attended more than one type of service.

^b^ Corresponding service type and numbers broken down below.

Interviews were conducted either in person or online between May of 2022 and May of 2023 and lasted between 60 to 90 min. Interviews followed a guide that explored trajectories, patterns and narratives of chemsex practice. Participants were asked about their treatment goals and motivation to access services. Participants were also asked several follow‐up questions about their histories of service use, recent and ongoing service use and treatment experiences. Interview participants received a $50 voucher to remunerate them for their participation.

### 2.3 Data analysis

Data from interviews were professionally transcribed verbatim and imported into NVivo 14. Data were analysed in accordance with a qualitative description methodology as outlined by Neergaard and Sandelowski [35, 36]. Qualitative description is a research methodology that has been used to examine experiences and practices in healthcare settings and an appropriate methodology for gathering perceptions from key stakeholders about poorly understood experiences [35].

In alignment with qualitative description methodology, the first author, Jack Freestone, familiarised himself with the data by reading transcripts prior to coding. Throughout reading Jack Freestone recorded his insights and reflections on the data using NVivo memos. He then re‐read the data to identify patterns and to inductively code themes using NVivo nodes and sub‐nodes. Identification and categorisation of themes was led by Jack Freestone and corroborated by Bo Justin Xiao. Together Jack Freestone and Bo Justin Xiaointerpreted the data to describe and devise the key findings presented below. Other authors also supported data interpretation, Adam Bourne, Krista J Siefried, Mohamed Hammoud and Garrett Prestage reviewed excerpts of data at a later stage of analysis and supported the early stage of write up.

## 3 RESULTS

Most participants (*n* = 21) were cisgender men who also identified as gay. One person was non‐binary; however, this person did not report any prior use of services to address drug use. All participants reported crystal methamphetamine use within the past 3 months and 19 reported recent injecting. Fewer participants (*n* = 15) reported recent GHB use.

As is outlined in Table 1, a total of 24 people were interviewed. Of these, 12 had sought services from counsellors or psychologists described as providing support for the general adult population. Mostly, these services were mental health services provided privately by counsellors or psychologists, given that these services are not specific or tailored to the needs of a particular population, we describe them in this paper as ‘mainstream services’. A total of 14 participants had engaged with ‘specialist services’. These are defined as specialist services because they were predominantly provided in locations where over 20% of the adult male population are estimated to be gay or bisexual men [37] and are known to have a high caseload of GBMSM practicing chemsex [13, 38]. Participants who had received ‘specialist services’ had attended either ACON's Substance Support Counselling service (*n* = 5), the St Vincent's Hospital Stimulant Treatment program (*n* = 6) or counselling in connection with an inner‐city sexual health service (*n* = 7) of LGBTQ+ community organisation (*n* = 1). ACON's Substance Support counselling services provides up to 12 free counselling sessions for LGBTQ+ people seeking to address their substance use, most substance support clients are GBMSM seeking to address their use of crystal methamphetamine [13]. St Vincent's Hospital Stimulant Treatment Program is an outpatient service, operating in Darlinghust, Sydney that offers free counselling and support for people who use stimulants, such as methamphetamine [39]. Publicly funded sexual health services in Australia offer routine sexual health screening and treatment but also provide counselling support for clients who may require additional support for matters relating to their sexual wellbeing [40]. Both the mainstream and specialist services referred to throughout this paper are outpatient talk therapies provided on a one‐to‐one rather than group basis.

A total of eight participants reported that they had not accessed counselling or psychologist services for their drug use. In four instances participants had not accessed services because they reported few harms associated with their drug use. In one instance a participant had not accessed services because of his involvement in an ongoing court case and his fears of legal ramifications. The remaining three participants spoke to geographic barriers associated with service access or lacking knowledge of appropriate support options.

Our findings are detailed below in three interlinked sub‐subsections. The first pertains to how our participants spoke about their treatment goals and motivations. The second addresses our participants reflections on barriers to achieving these goals and the third includes reflections on participant's service experiences.

### 3.1 Treatment goals

When articulating their treatment goals, nearly all participants sought to reduce their use of crystal methamphetamine and described as occurring in the context of chemsex. Although treatment goals were discussed with regards to patterns of crystal methamphetamine use, the motivators underlying these goals related to improved wellbeing, mental health, relationships and social connection. At the time of accessing services, many participants had come to recognise detrimental impacts of chemsex on relationships, social engagement or mental health or were concerned about escalations relating to chemsex frequency, amount of drugs consumed and durations of a singular occasions of use. In some circumstances participants were motivated to access services to prevent a financial, housing or employment crisis framed as imminent and threatening. The relationship between treatment goals, the motivations behind these goals and the trigger to access a service is demonstrated in the following quote from Michael.

‘I had a big overdose from injecting. And that was then I realised the pivot, I realised now I needed to go and get help. “I need to take a break from this.” And I think I just had to stop 'cause, if I don't, then I feel like it's gonna, I'm gonna get worse and worse, and I'm just gonna get to a point where I'll be injecting three points. And then that's when I'll be, I'll be down that rabbit hole, and I won't be able to get out. And I'll be forever addicted to it… So, since then, it's been two months since I haven't used any’. Michael, 20s

Unlike Michael, a small number of participants who had accessed services did not seek to reduce crystal methamphetamine use but wished to manage harms relating to chemsex by changing their method of administration or reducing the duration of chemsex sessions or introduce stronger boundaries around their sexual partner selection.

### 3.2 Challenges associated with achieving treatment goals

The task of reducing crystal methamphetamine use presented multiple challenges. These were said to include disengagement with relationships, learned reliance on crystal methamphetamine to cope with boredom, stress and assuage low mood. However, participants also emphasised a sense of ambivalence, trepidation, and worry about their inability to have sex without using drugs and spoke about sex as a primary driver of their drug use. The linked and bi‐directional relationship between crystal methamphetamine and sex was articulated by several participants. For example, Lisandro articulated how routine sexual stimulus triggered his drug use:

‘For me the thing with meth is it's always related to sex. I don't do it for other purpose. Yeah. Porn, sex … one thing is related to another. And sometimes those are the triggers … Like, you know, I see a hot guy on the TV. You say, “Oh!” you know. It comes to my mind’. Lisandro, 30s

Similarly, Sebastian acknowledged his human need for sex and articulated this need in opposition to his treatment goals of reducing drug use.

‘I always used to say a counsellor, “If I can learn to have sex sober, my battle has been won,” because then I don't need the drugs, 'cause I'm using the drug to have sex. And sex is a human … To want to have sex is a human need’. Sebastian, 40s

Sebastian narrated having sex sober as a skill that he could learn and framed this learning as key to his ability to manage his drug use. Other participants who spoke about the centrality of drugs to their sex lives framed drugs as central to their sexual identities, behaviours and pleasure. These sentiments were reflected by both Oscar and Dimitri.

‘In the past three years, I can probably count on one hand the amount of times I've had sober sex. And I'm someone who has a lot of sex … And the only reason why I inject is because one of the sex acts I enjoy is fisting, and I find it impossible to do without injecting …. You know when you have drugs it numbs you to a certain extent. Which means two things: it means that the duration of time that you can bottom is almost infinite … and the second thing is the intensity of that fucking is also almost infinite, in terms of what you can tolerate. And so, because many of my regulars now that's how they know me, right, there's no way I could be like that sober’. Dimitri, 40s

For Dimitri, a departure from chemsex was thought of as a departure from sexual possibilities and sexual networks. Dimitri spoke about his reputation for sexual duration and intensity within his sexual network and alluded to the pressures of expectations around the intensity of a sexual performance enabled by injecting. Dimitri seemed to fear the loss of connection to his sexual network however, for Jason a reduction of chemsex resulted in the loss of intimacy within his romantic relationship. Jason expressed grief around this experience:

‘One of the problems with my ex was that, when I was sort of weaning us off usage, then like I found it difficult to be physically intimate with him, which was a terrible thing to realise’. Jason, 60s

The inextricability of drugs from participants' sexual practices and identities and the ways in which drug use was narrated as enabling sexual pleasure and participation in community was commonly spoken about as a source of ambivalence, distress and confusion. To achieve their treatment goal of reducing their frequency of crystal methamphetamine use and by extension their practice of chemsex, our participants seemingly required counsellors and psychologists to appropriately explore the varied sexual drivers and experiences incumbent in their drug use.

### 3.3 Experiences of counselling and psychology services

No participants in our study reported experiencing overt homophobic discrimination within one‐to‐one counselling or psychology services, however participants reported ongoing anticipated and perceived stigma as well as internalised or self‐stigmas relating to their drug use, the sexual drivers of their drug use and their sexual practices. These stigmas were particularly felt within mainstream mental health and drug services and in these settings participants reported the censorship of their sexual desires. In most instances providers did not ask participants about sex but rather focussed on drug use or mental health as indicated by Robbie.

‘When I think of the biggest reasons why I find it hard to give up is not because I want to have drugs every weekend. It's like, “Oh, but, if I'm not having drugs, I won't have sex.” So, there's no conversation about the, sex as a trigger in itself. Like it was very related to drug use. And I mean, yes, drugs are the health issue, not sex. I don't know. I just always find for me the thing that I don't understand and what I need help with is talking to someone about how I get confidence to have sex when, when not on drugs, and stuff like that … I find whenever you ask for help it's, it's solely about reducing drug use. But they go hand in hand’. Robbie, 30s

Participants commonly reported that they had gleaned several benefits from interactions with counsellors and psychologists. Benefits included improved mental health, self‐awareness, the ability to recognise and manage triggers, greater social connection and referrals to other supportive services. Reportedly participants did not feel able to speak about the sexual drivers, contexts and practices that reinforced their drug use and instead opted to work with providers on health and wellbeing priorities outside of chemsex. Multiple barriers to disclosure around sex within talk therapies were reported. Most of the time barriers appeared to relate to participants' perception of a provider's openness, their sexuality, HIV status and their prior lived experiences in terms of drug use, and chemsex specifically.

Robbie narrated an inability to disclose details of his chemsex (he refers to chemsex as PnP) to a psychologist who he perceived as not knowing much about chemsex.

‘I don't think he was aware of or could go into much detail of what PnP was and, therefore, I didn't feel comfortable going into detail. Yes, I could say I've taken a lot of drugs but I'm not going into detail of what that situation was …even with a psychologist it's hard to be open about it, something that's got like a lot of taboo around it, yeah …. I had other issues other than just PnP to work through, and I found him like probably, like one of the, like the best psychologist I've ever been to. Like really, really good’. Robbie, 30s

Robbie used the term taboo to describe chemsex and his anticipation of chemsex stigma and a perception that his therapist would be judgemental prevented disclosure around chemsex.

Like Robbie, several participants spoke about feeling uncomfortable with open discussion with psychologists perceived as lacking lived experience of chemsex. One such participant was Luca who reflected:

‘You'll speak to psychologists and they're just … They don't have that lived experience, so it's very difficult for them to kind of relate to and the topic itself is quite shocking, right? To be like, “Do you know what? I wanna get like loaded up on this stuff and then get railed by 14 guys while they do X, Y and Z.” Like that's something that's really difficult to have a conversation with someone about, and, so like sharing that with a psychologist can be very difficult … When I'm talking about the drug use with service providers, I'm talking about the use. I'm not talking about the activities while the use is happening because if you think that drug use is shameful, let me introduce you to the kind of things that you do while you're on say drugs. So, like shame times a thousand’. Luca, 30s

Luca used the word ‘shocking’ to describe chemsex and spoke of shame around drugs use as compounded by his sense of shame around adventurous sex. Like Luca, several participants framed their sexual behaviours, their injecting behaviours, their loneliness and experiences of loss around employment or relationships as embarrassing or shameful. For some, their internalisation of stigmas around these practices or experiences contributed to a low self‐esteem which prevented disclosures.

Another barrier to disclosures around the sexual contexts and drivers of drug use within talk therapies provided in the context of alcohol and other drug (AOD) services, appeared to be the perception that the remit of AOD services was limited to AOD use. This perception was demonstrated by Desmond who said:

‘We probably didn't even talk about any of the sex side of stuff at all, or the situations that probably that drug use, in terms of partying and sex, has gotten myself certainly into …’

JF: ‘Was the sex side of stuff something that you wanted to talk about?’

‘With the counselling? Potentially, yeah. Like, potentially, I think it would have been because I think, yeah, potentially’.

JF: ‘Do you have any thoughts about why the sex stuff didn't come up in those sessions?’

‘I really don't even know why it didn't come up, to be honest. I think it was probably more around, it was really just focused on substance abuse. And I don't think it really probably encapsulated the breadth that, you know, drug or substance abuse impacts all areas of peoples' lives. Obviously, interpersonal relationships and sex’. Desmond, 40s

Upon being prompted, Desmond acknowledged that a conversation about sex when seeking counselling at an AOD service may have been beneficial. However, prior to being prompted Desmond had not conceived that it may have been useful, possible or appropriate to discuss sex at an AOD service.

As noted in Table 1, 14 participants addressed chemsex with counsellors or psychologists within specialist services. Of these 14 participants, 10 had previously also attended mainstream providers and these participants commonly sought out specialist services to specifically address chemsex. This experience is reflected in the quotes below from Oscar. Oscar first described his experience within a mainstream service and second described his transition to a specialist chemsex service.

‘They say it's confidential but how confidential? … How, how open can you be with somebody like that? I still think they judge’.

‘Everyone was friendly. There was no judgement. It was just walk in, say what, say, you know, what you're here for and then openly discuss things that aren't discussed or things that are frowned upon by anyone else’. Oscar, 50s

Several participants like Oscar had sought out specialist chemsex services and these participants indicated that a reputation of speciality at a service level alleviated concerns about judgement at an individual provider level. Service reputability was seemingly reinforced by positive community feedback, a perception of or understanding of the service as either an LGBTQ+ specific service or LGBTQ+ inclusive service and the perception of service providers as peers in terms of their sexuality, gender and lived experience of drug use.

Some, but not all, participants who had engaged specialist providers said that they had been supported to address experiences of isolation, sexual shame, sexual identity, relationships, chemsex trigger management and the relationship between substance use and sex.

Some of these experiences are reflected in the quotes from Sam and Richard:

‘Talked about I guess reasons why people use drugs in general and sex and drugs… because I think a lot of my anxiety to do with dating and sex, it's around this concept of people don't wanna be with me or whatever. Whereas with the drugs I guess like they're kind of there because the drug is making them or whatever. So, there's been lots of things we've covered, and I guess it's been useful’. Sam, 30s

‘I suppose he wanted to explore that area of sexuality. And, so, you know, chicken and the egg stuff. So, what comes first? Is it you use drugs to get off or is it you get off and then you use drugs? And so, he wanted to explore that’. Richard, 60s

As is indicated in Sam and Richard's quotes, the therapeutic action around addressing the relationship between sex and drugs seemed to centre personal reflections and awareness raising. Perspectives from multiple participants indicated that a providers' proactive engagement in discussions about sex enabled relevant support and mitigated anticipated, perceived and self‐stigma around sex.

## 4 DISCUSSION

Prior studies among GBMSM practicing chemsex [11] and broader populations of people who use drugs [41] have noted that anticipated and perceived stigmas prevent service access. In our study, participants continued to anticipate and perceive stigma throughout engagement with services and avoided enacted stigma through non‐disclosure. Importantly, our participants did not appear to censor their use of drugs, and they did not appear to anticipate or perceive stigmas associated with their sexuality. Rather, participants anticipated and perceived stigmas associated with their sexual desires, sexual behaviours and their use of drugs to facilitate sex.

Among our participants self‐censorship appeared to be pronounced when interacting with mainstream AOD or mental health services. Although participants acknowledged the beneficial supports these services offered, many felt compelled to address chemsex within services with known chemsex expertise and broader LGBTQ+ cultural competence. At a high level, cultural competence refers to the provision of acceptable and effective healthcare for people from diverse cultures and communities [42], there are guidelines to support the delivery of inclusive affirming AOD services for LGBTQ+ populations [43]. Our findings echo prior recommendations regarding the service offering for GBMSM who practice chemsex, these being that GBMSM require inclusive mainstream services [13, 20, 33, 44, 45] alongside services with tailored expertise [13]. Recommendations also suggest that services offer robust referral pathways to specialist providers [43] and that services provide interdisciplinary care at the intersection of drug use, mental health, sexual health and sexual wellbeing [20]. Several participants in our study reported receipt of affirming counselling services at sexual health clinics, echoing findings of previous research that has highlighted sexual health clinics as promising sites for chemsex interventions [12, 26, 46].

There is extensive commentary in the empirical evidence to support the delivery of peer‐led chemsex interventions [45, 47, 48] and several examples of peer‐led brief interventions and group programs operating within Australia and internationally [26, 49, 50, 51]. In the broader literature on peer‐led interventions for GBMSM, peers, by virtue of their reciprocal lived experiences or identities are hypothesised to break down barriers to care, deliver culturally competent interventions and achieve therapeutically beneficial rapid rapport [48]. Although the focus of this study was on services received by counsellors and psychologists with clinical qualifications, our findings indicate that the perception that professionals lacked lived experience and cultural competence, prevented them from speaking about the sexual drivers of their methamphetamine use. Our findings concur with recommendation that effective chemsex interventions may be delivered by peers [12, 47].

Participants in our study spoke about their internalisation of societal stigmas around their drug use and incumbent sexual practices which amounted to a shame that reinforced self‐censorship. Reluctance among therapists to discuss sex with their clients and a dual reluctance among clients to discuss sex with their therapists is well documented [52, 53]. This inhibition around discussing sex is troublesome because sex is a central tenet of psychological health, sexual health and wellbeing. Sex ought to be actively addressed within talk therapies regardless of one's experience of sexual distress or dysfunction [27, 52]. However, the evidence base on the use of drugs for sexual functioning and treatment approaches to support sexual wellbeing is scant. More studies on treatment approaches that may support people around their ability to have sex without drugs and their overall sexual wellbeing are required. Some practitioners have explored treatment approaches to support people practicing chemsex to reengage with sober sex and have detailed the use of mindfulness [31], masturbation [32] and tailored psychoeducation approaches [54]. These approaches provide some preliminary indication around treatment strategies that may potentially be effective but further research is required.

Our findings must be interpreted with acknowledgement of several limitations. Notably, prior access to either counselling or psychology services was not a criteria for participation and eight of our participants reported that they had not previously engaged these services. Further, we recruited many of our participants via promotion though ACON and St Vincent's Stimulant Treatment Program. We did not recruit directly from mainstream services, therefore we did not sample GBMSM who may be well served within mainstream services and who do not seek specialist services. Further, our eligibility criteria were limited to people currently engaged in sexualised GHB or crystal methamphetamine use, therefore our findings do not represent the experiences of people who are currently abstinent. Drawing on the recommendation that services provide inter‐disciplinary chemsex support, our data collection and analysis explored service experiences at the intersection of sexual wellbeing and drug use. This important focus prohibited a wider focus on several other topics that are worthy of exploration, such as whether other stigmatised behaviours such as injecting were addressed by counsellors and psychologists. Our eligibility criteria included trans women and non‐binary people however no trans women were interviewed and only one non‐binary person was interviewed. Furthermore, our sample lacked significant cultural diversity, most of our participants were white GBMSM and we did not interview any Aboriginal or Torres Strait Islander people. Finally, most of our data pertained to the receipt of talk therapies in outpatient settings and nearly all of our participants sought to reduce their practice of chemsex. Therefore, this study does not include insights around support for clients around harm reduction, nor does it address experiences within residential services, emergency or general practice. Further research is required to generate a more nuanced understanding of treatment needs and experiences across a diverse range of treatment modalities.

## 5 CONCLUSIONS

Our findings underscore an imperative for counsellors and psychologists to proactively ask about and explore the relationship between sex and drug use when working with GBMSM who practice chemsex. The perspectives shared by GBMSM underscore a need for therapeutic approaches to alleviate sexual shame and explicitly address one of the major barriers to chemsex reduction; a person's inability to have sex without drugs. Unfortunately, psychologists and counsellors often receive limited training on sex and may lack confidence in this sensitive, complex and value laden arena [52, 55]. Our study highlights that investment in training and supervision around sexual therapies for those working alongside GBMSM who practice chemsex may be beneficial. Our findings also affirm the need for peer‐led services and for mainstream services to develop strong partnerships and referral pathways with specialist providers.

## AUTHOR CONTRIBUTIONS

Conceptualisation and design of this research was supported by all authors. Jack Freestone led data collection and led analysis in collaboration with Bo Justin Xiao. Jack Freestone also drafted and revised this manuscript, while Adam Bourne, Krista J Siefried, Mohamed Hammoud and Garrett Prestage conducted a review of the first draft of this manuscript and supported with data interpretation. Robert Stirling, Nadine Ezard, Rick Varma, Louisa Degenhardt, Lisa Maher, provided support at the stage of research conceptualisation and provided additional and significant contribution throughout manuscript revision and editing.

## CONFLICT OF INTEREST STATEMENT

For the duration of this study, the first author Jack Freestone and the second author Bo Justin Xiao were employed at ACON, a community organisation that receives funding to deliver tailored services to lesbian, gay, bisexual, transgender and queer communities in New South Wales Australia. The first author was employed by ACON while he conducted this research. As indicated in the text, ACON is, a community organisation that specialises in health promotion and clinical services for sexuality and gender diverse communities in New South Wales. This research was funded by an NHMRC Partnership Grant.

## Data Availability

Research data are not shared.
